# An improved assay for rapid detection of viable *Staphylococcus aureus* cells by incorporating surfactant and PMA treatments in qPCR

**DOI:** 10.1186/s12866-018-1273-x

**Published:** 2018-10-11

**Authors:** Chen Zi, Dexin Zeng, Nan Ling, Jianjun Dai, Feng Xue, Yuan Jiang, Baoguang Li

**Affiliations:** 1 MOE Joint International Research Laboratory of Animal Health and Food Safety, College of Veterinary Medicine, Nanjing Agricultural University, Nanjing, 210095 China; 2 Shanghai Entry-Exit Inspection and Quarantine Bureau, Shanghai, 200135 China; 3 Division of Molecular Biology, Center for Food Safety and Applied Nutrition, U.S. Food and Drug Administration, Laurel, MD 20708 USA

**Keywords:** *Staphylococcus aureus*, PMA-qPCR, Sarkosyl, Triton x-100, Accurate detection, Viability, False positive, Milk, Methicillin-resistant *Staphylococcus aureus*

## Abstract

**Background:**

*Staphylococcus aureus* is an important human pathogen causing a variety of life-threatening diseases. Rapid and accurate detection of *Staphylococcus aureus* is a necessity for prevention of outbreaks caused by this pathogen. PCR is a useful tool for rapid detection of foodborne pathogens, however, its inability to differentiate DNA from dead cells and live cells in amplification severely limits its application in pathogen detection. The aim of this study was to develop an improved assay was developed by incorporating the sample treatments with a surfactant and propidium monoazide (PMA) in qPCR for detection of viable *S. aureus* cells.

**Results:**

The cell toxic effect testing with the two surfactants showed that the viability of *S. aureus* was virtually not affected by the treatment with 0.5% triton x-100 or 0.025% sarkosyl. Triton x-100 was coupled with PMA for sample treatments for detection of viable *S. aureus* cells in artificially contaminated milk. The qPCR results indicated that the assay reached high an amplification efficiency of 98.44% and the live *S. aureus* cells were accurately detected from the triton-treated spiked milk samples by the PMA-qPCR assay.

**Conclusions:**

The qPCR assay combined with treatments of PMA and surfactants offers a sensitive and accurate means for detection of viable *S. aureus* cells. Cell toxic effect testing with the two surfactants showed that the viability of *S. aureus* was virtually not affected by the treatment with 0.5% triton x-100 or 0.025% sarkosyl. The information on sample treatment with surfactants to improve the dead cell DNA removal efficiency in qPCR by increasing PMA’s permeability to dead cells can be used for other pathogens, especially for Gram-positive bacteria.

## Background

*Staphylococcus aureus*, a Gram-positive bacterium, is an important human pathogen causing various life-threatening infections [1]. Antibiotic resistance resulted from long-term use and misuse of antibiotics causes people to be sick for longer and increases the risk of death. Methicillin-resistant *Staphylococcus aureus* (MRSA) is an important and growing cause of staphylococcal infection [2–4]. A report from World Health Organization (WHO) showed that it is 64% more likely to die for people infected by MRSA than those by non-resistant form [5]. Thus, it is imperative to have to ample availability of methodologies for rapid and accurate detection of *S. aureus* to protect the food supply chain and curtail misuse of antibiotics.

PCR has become a common and useful technology in detection of foodborne pathogens and greatly enhanced the efficiency of pathogen detection. However, DNA can persist for long period of time in the environment even after cells’ death; and the residual DNA could not be completely eliminated by high temperature (121 °C for 15 min) [6]. Consequently, the DNA from the dead cells may be amplified in PCR reaction. Thus, PCR’s inability to differentiate DNA from dead cells and live cells in amplification constitutes a serious drawback to its application in pathogen detection [7].

To remedy this shortcoming of PCR, there are a few options. One practical approach is the use of a biological dye, propidium monoazide (PMA) [7]. Treatment of cells with ethidium monoazide (EMA) or PMA (a derivative of EMA) has been used in conjunction with qPCR (EMA/PMA-qPCR) to distinguish live and dead cells using membrane integrity as viability criterion [8]. The viability discrimination is based on the characteristics of the dyes: EMA and PMA. EMA or PMA is positively charged molecule, and thus is excluded by intact, negatively charged bacterial cell-membranes, but can enter bacteria with compromised cell-membranes. When they selectively enter the compromised cells, the dye intercalates into nucleic acids and forms a covalent modification between the dye and DNA after exposure to bright visible light [9]. Thus, PCR can preferentially amplify the DNA of viable cells. Researchers have showed that PMA was more selective than EMA in inhibiting DNA amplification from dead cells [10]. PMA has been widely used in conjunction with PCR to limit false-positive PCR results in detection of foodborne pathogens, especially with Gram-negative bacteria such as *Escherichia coli*, *Salmonella* [10, 11]. However, little is known about PMA’s permeability to the cell-membranes of dead *S. aureus* cells. It has shown that detergents can improve PMA or EMA’s permeability to dead cells without compromising the viability of live cells [12]. Sarkosyl, a surfactant, has been used for dissipation of PMA-barrier properties of membranes of inactivated *E. coli* cells [13]; and triton x-100, another surfactant, has been tried to increase the permeability of bacteria. In this study, we evaluated the efficiency of the two surfactants in improving PMA’s permeability to dead *S. aureus* cells and combined the surfactant and PMA treatments with qPCR to improve the dead cell DNA removal efficiency. Moreover, we have applied the PMA-qPCR for rapid and accurate detection of live *S. aureus* cells in spiked milk.

## Methods

### Bacterial strains and culture conditions

The *S. aureus* (ATCC25923), a reference strain used throughout the study, was inoculated in Luria Bertani (LB) and incubated at 37 °C shaking for 18 h. The bacterial culture was centrifuged at 8000×g for 10 min and washed thrice with phosphate buffered saline (PBS), then the bacteria were resuspended in PBS. The bacterial cells were diluted 10-fold (up to 10^7^) and plated onto LB agar plates for viable cell count. The diluted cell suspensions were equally divided into two sets of aliquots. One set of the aliquots was heated at 100 °C for 20 min for dead cells, and the other set was not heated for live cells. All other strains used in the inclusivity and exclusivity tests were grown in the same conditions as the strain of *S. aureus* (ATCC25923) and listed in Table 1.

**Table 1:** Bacterial strains used for the inclusivity and exclusivity tests in this study

Bacterial species	Strain	Source	Detection result by qPCR
*Staphylococcus aureus (n = 20)*	ATCC 25923	ATCC^a^	+
	SA1011 #	Flour product	+
	SA1012 #	Pork	+
	SA1013 #	Frozen corn	+
	SA1014 #	Raw milk	+
	SA1015 #	Raw milk	+
	SA1016 #	Raw milk	+
	SA1017 #	Human (sputum)	+
	SA1018 #	Human (blood)	+
	SA1019 #	Human (pus)	+
	SA1020 #	Human (wound)	+
	SA1131 #	Human (sputum)	+
	SA1132 #	Flour product	+
	SA1133 #	Pork	+
	SA1134 #	Frozen corn	+
	SA1135 #	Raw milk	+
	SA1136 #	Pig	+
	SA1137 #	Pork	+
	SA1138 #	Pig	+
	SA1139 #	Raw milk	+
	SA1140 #	Raw milk	+
*Salmonella Typhimurium*	ATCC 14028	ATCC	–
*Salmonella Typhimurium*	CICC21484	CICC^b^	–
*Salmonella Enteritidis*	CICC24119	CICC	–
*Escherichia coli*	ATCC 25922	ATCC	–
*Listeria monocytogenes*	CMCC54002	CMCC^c^	–
*Listeria grayi*	ATCC 25401	ATCC	–
*Listeria seeligeri*	ATCC 35967	ATCC	–
*Listeria welshimeri*	ATCC 35897	ATCC	–
*Listeria ivanovii (n = 7)*	ATCC 19119	ATCC	–
	CMCC89001	CMCC	–
	VP61 #	Human (anal swab)	–
	VP65 #	Restaurant	–
	VP67 #	Trout	–
	VP105 #	Bass	–
	VP106 #	Clams	–
*Vibrio parahemolyticus*	VP108 #	Cuttlefish	–
*Streptococcus hemolyticus*	ATCC 21059	ATCC	–
*Campylobacter jejuni (n = 4)*	ATCC 33291	ATCC	–
	ATCC 43464	ATCC	–
	CJ2 #	Human	–
	CJ12 #	Chicken	–
*Clostridium perfringens*	ATCC 13124	ATCC	–
*Bacillus cereus*	ATCC 11778	ATCC	–
*Pseudomonas aeruginosa*	ATCC 27853	ATCC	–

^a^ATCC refers to American Type Culture Collection, USA

^b^CVM-NAU refers to College of Veterinary Medicine, Nanjing Agricultural University, Nanjing, China

^c^CMCC refers to National Center for Medical Culture, China

### Sample treatments with triton x-100 and sarkosyl

To test the cell toxicity of triton x-100 and sarkosyl to *S. aureus* cells, different concentrations of the two surfactants (0.1%, 0.25%, 0.5%, and 2% triton x-100; 0.005%, 0.025%, 0.05%, and 0.25% sarkosyl) were added to 1 ml of bacterial suspension as shown in Table 2. After incubation at 37 °C for 20 min, the treated bacteria were 10-fold serially diluted. The cell dilutions were plated onto LB agar plates and incubate at 37 °C for 18 h for viable cell count.

**Table 2:** Toxic effect of the cell treatment with two surfactants to live *S. aureus* cells assessed by colony count

Surfactant	Triton x-100 concentration (v/v)				Sarkosyl concentration (m/v)				NC
	0.10%	0.25%	0.50%	2%	0.005%	0.025%	0.05%	0.25%	
CFU/mL^a^	4.64 × 10^6^	4.68 × 10^6^	4.63 × 10^6^	**3.57 ×106** ^**b**^	4.77 × 10^6^	4.62 × 10^6^	**1.57 × 10** ^**5**^	**1.02 × 10** ^**3**^	4.4 × 10^6^

^a^refers to the mean of colony counts in three plates

^b^bold-faced number refers to a significant difference (*p* < 0.01) compared to the negative control (NC) or PBS control

### Optimization of PMA concentration for cell treatment

Based on the effect of surfactant treatment to the viability of *S. aureus* cells, bacterial cells were treated with appropriate concentration of two surfactants (0.5% triton x-100 or 0.025% sarkosyl) for 20 min. Then, the cells were treated with PMA (Biotium, USA). PMA was dissolved in DMSO to 10 mM solution and stored at − 20°. To determine the optimal concentration for PMA treatment, different concentrations of PMA (1, 4, 10, 40, and 100 μM) were tested. Briefly, 1 ml of *S. aureus* cells (10^6^ CFU/ml) were incubated with different concentrations of PMA for 5 min in the dark, and then the cells were exposed to an intensive light source (BLU-V System, Qiagen, Germany) for 15 min. The bacteria were collected by centrifugation at 8000×g for 10 min, and then proceeded for DNA extraction with a TIANamp bacterial DNA kit (Tiangen Biotech).

### Setting the qPCR assay conditions

Primers and TaqMan probe were designed based on the sequences of GenBank (Accession number, CP019563.1) using Primer Premier 5.0 program (forward primer, TTCGCTACTAGTTGCTTA; reverse primer, GCACTATATACTGTTGGATC; and probe, FAM-TCAGAACCACTTCTATTTACGCCGT-TAMARA). The expected length of PCR amplicon was 124 bp. The PCR reaction mix was consisted of 10.0 μl of Premix Ex Taq Master Mix (Takara, Dalian, China), 200 nM forward and reverse primers, 200 nM probe, 200 nM ROX Reference Dye II (Takara, Dalian, China), 5 μl of template DNA, and 3.4 μl of water to make up 20 μl as the final reaction volume. The qPCR amplification was initially started at 95 °C for 15 s, then 95 °C for 5 s, and followed by 62 °C for 34 s (40 cycles). SPSS Statistics 17.0 (software) was used to analyze significant difference between different groups by the method of General Linear Model (GLM).

### Sensitivity test

A serial of 10-fold dilution (10^1^–10^6^) of *S. aureus* cell culture was made, and DNA extraction of different dilution was performed with a TIANamp bacterial DNA kit. The resultant DNA was analyzed by qPCR.

### Inclusivity and exclusivity tests

*S. aureus* strains (*n* = 21), including the reference strain (ATCC25923) were tested for inclusivity test. The exclusivity test covered non-*S. aureus* strains (*n* = 30), including common foodborne pathogens (Table 1).

### Detection of live cells in mixture of live and dead cells

Dead *S. aureus* cells (10^5^) were mixed with 10^2^, 10^3^, 10^4^, and 10^5^ viable cells, respectively, and 0.5% triton x-100 was also added into each sample. The cell mixtures were split equally into two aliquots. One set of the aliquots was treated with 40 μM PMA, and the other set was not treated. After the PMA treatment, both the PMA-treated and untreated cell mixtures were subjected to DNA extraction. Subsequently, the DNA was analyzed by qPCR as mentioned about.

### Detection of viable cells of *S. aureus* in spiked milk

To assess the ability of the PMA-qPCR assay in distinguishing viable and dead cells in food, we applied this assay for detection of viable *S. aureus* cells in spiked milk. Pasteurized whole milk was purchased from a local food market and confirmed to be free of *S. aureus.* Each milk sample (1 ml) was mixed with 1.0 × 10^4^ dead *S. aureus* cells and different amount of live *S. aureus* cells (10^2^, 10^3^, 10^4^, 10^5^, and 10^6^ CFU/ml), respectively. Each concentration was sampled in triplicate. Then the spiked milk samples were centrifuged at 8000×g for 10 min, and the cream and liquid in the tube were removed. The cell pellets were washed three times with PBS, and then 1 ml of PBS with 0.5% triton x-100 was used to resuspend the pellets. The cell resuspensions were incubated at 37 °C for 20 min. The cell samples were treated with 40 μM of PMA in the dark and exposed to intensive light. DNA extraction and PMA-qPCR were performed in the same methods as mentioned above. The live cell control was treated in the same way except that no PMA was used.

### Statistical analysis

All statistical analyses were performed using SPSS17.0 software.

## Results

### Sensitivity and specificity of the qPCR assay

**Fig. 1:**
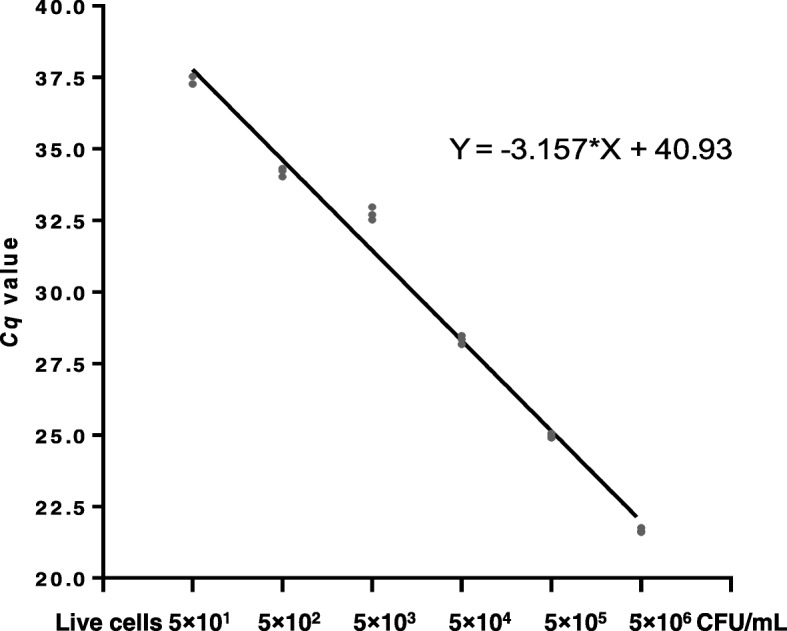
Performance of detection of *S. aureus* by the qPCR assay*.* The standard curve showed a R^2^ of 0.99, a slope of − 3.16, and an amplification efficiency of 100.36%. The *Cq* values of serially diluted cell suspensions (5.0 × 10^7^, 5.0 × 10^6^, 5.0 × 10^5^, 5.0 × 10^4^, 5.0 × 10^3^, 5.0 × 10^2^, 5.0 × 10^1^), indicating a LOD of 5.0 × 10^1^ *S. aureus* cells with a *Cq* value of 37.36

The qPCR assay generated a standard curvet with a slope of − 3.16 and amplification efficiency of 100.36%. The R^2^ is extremely close to 1.0, presenting a robust PCR amplification (Fig. 1). The result of qPCR assay with the serially diluted cell suspension showed a *Cq* value of 37.36 on the sample of 5.0 × 10^1^ *S. aureus* cells.

All the *S. aureus* strains tested (*n* = 21) were positively detected by the qPCR assay; and all the non-*S. aureus* strains tested (*n* = 30) were shown negative and no cross-reactivity was detected with the non-*S. aureus* strains (Table 1).

### Cells treated with different concentrations of triton x-100 and sarkosyl

The two surfactants (triton x-100 and sarkosyl) used to treat *S. aureus* cells in this study demonstrated various degrees of effect to the cell viability by different concentration of surfactants as shown in Table 2. For example, the viability of *S. aureus* cells treated with triton x-100 was not affected when the concentration was 0.5% or less (0.1%, 0.25, and 0.5), while the cell viability was significantly affected (*P* < 0.01) by 2% triton x-100 (Table 2). Similarly, different concentration of sarkosyl demonstrated various degrees of effect on the cell viability. For example, a vast majority of the cells lost viability when treated with either 0.05% or 0.25% sarkosyl for 20 min; however, there was negligible effect to the cell viability when the concentration of sarkosyl was 0.025% or less (Table 2). Based on these data, 0.5% triton x-100 and 0.025% sarkosyl should be chosen for sample treatment.

### Optimization of the PMA concentration for cell treatment

The PMA-qPCR results showed similar trends between the dead *S. aureus* cells treated with PMA in conjunction with treatment of triton x-100 or sarkosyl (Table 3). In general, the higher concentrations of PMA, the more efficient in preventing amplification of the DNA of dead cells. As the PMA concentration increased, the *Cq* values of the dead cells went higher (higher removal efficiency of dead cell DNA). The groups treated with triton x-100 or sarkosyl showed significantly higher *Cq* values (about 2 *Cq* values) compared with the control group (PBS) as shown in Table 3. Between the two groups treated with triton x-100 or sarkosyl, the *Cq* values of the sarkosyl-treated group were higher than those of the triton x-100-treated group with high concentration of PMA (> 10 μM). For the viable cells, on the other hand, the *Cq* values of the triton-treated samples were significantly lower than those of the sarkosyl-treated samples, except for the 100 μM PMA treatment (Table 4), suggesting sample treatment with triton x-100 is milder to viable cells compared to sample treatment with sarkosyl. Based on the result of triton x-100 treatment, the *Cq* value of 40 μM PMA treated sample was significantly higher than the *Cq* values of the samples treated with PMA of 4 μM or 10 μM. However, there were no significant differences between 4 μM and 10 μM, nor between 40 μM and 100 μM (Table 3). Hence, we chose 40 μM of PMA for the optimal concentration in this study.

**Table 3:** Influence of the treatment of sarkosyl or triton × − 100 on the removal efficiency of the DNA of dead cells in the PMA-qPCR assay

PMA concentration (μM)	Sarkosyl (0.025%)	Triton × − 100 (0.5%)	PBS
4	**31.68 ± 0.35** ^a^ ^b^	**32.53 ± 0.20**	29.94 ± 0.33
10	33.40 ± 0.49	33.13 ± 0.56	31.58 ± 0.19
40	34.38 ± 0.46	33.64 ± 0.43	32.31 ± 0.31
100	**34.88 ± 0.46**	**33.88 ± 0.34**	33.20 ± 0.13
NC^c^	30.52 ± 0.52	29.79 ± 0.42	28.65 ± 0.35

^a^refers to the mean Cq value of triplicate ± standard deviation

^b^bold-faced number refers to a value of a sample treated with the same concentration of PMA that demonstrated a significant difference (*p* < 0.05) between the two surfactants used

^c^refers to negative controls, which were treated only with surfactant or PBS but without PMA

**Table 4:** The combination effect of the treatments of PMA with triton x-100 or sarkosyl on live *S. aureus* cells assessed by qPCR

PMA concentration (μM)	Triton x-100 (0.5%)	Sarkosyl (0.025%)
1	27.33 ± 0.12^a^	28.64 ± 0.13
4	**27.94 ± 0.04** ^**b**^	**29.10 ± 0.04**
10	**27.71 ± 0.25**	**29.65 ± 0.34**
40	**27.94 ± 0.04**	**29.54 ± 0.40**
100	28.76 ± 0.13	28.64 ± 0.13
NC^c^	**23.15 ± 0.10**	**26.24 ± 0.09**

^a^refers to the mean Cqvalue of the triplicates ± standard deviation

^b^bold-faced number refers to a value of a sample treated with the same concentration of PMA that demonstrated a significant difference (p < 0.05) between the two surfactants used

^c^NC refers to a negative control that was treated only with a surfactant but no PMA treatment

### Detection of the live cells in live and dead cell mixture

**Fig. 2:**
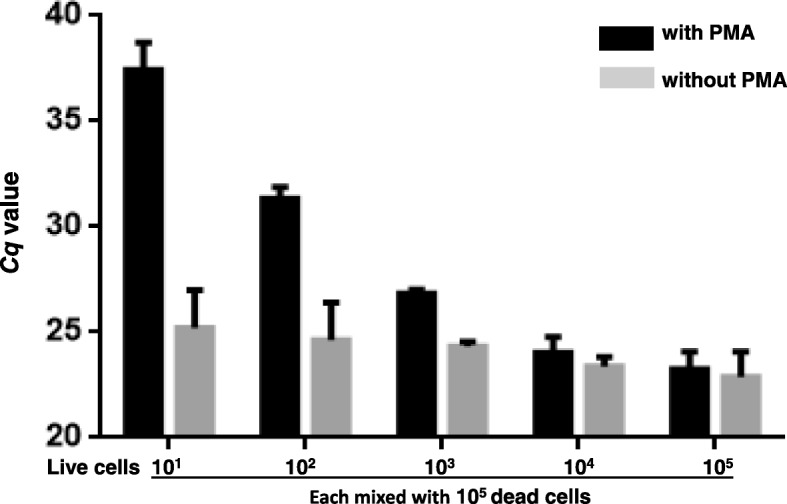
Detection of viable cells of *S. aureus* in the presence of a large number of dead cells by the PMA-qPCR. The numbers on the X axis (1–5) represent the number of live cells in the samples (0, 1.0 × 10^2^, 5.0 × 10^3^, 1.0 × 10^4^, and 2.0× 10^5^ CFU/ml, respectively), mixed with 2.0 × 10^5^ CFU/ml dead cells

The result of PMA-qPCR demonstrated a trend that the *Cq* values were getting lower in proportion with the increase of the viable cells after treated with triton x-100 and PMA in the presence of a large number of dead cells (Fig. 2). For instance, two samples (dead cells only; and dead cells with 10^2^ viable cells/ml) treated with or without PMA demonstrated drastic differences in the PMA-qPCR result, i.e., the PMA-treat samples showed *Cq* values of approximately 38 and 32; whereas the two samples when were not treated with PMA both generated *Cq* values approximately 25 (Fig. 2). These results indicated that the PMA-qPCR assay developed in this study is able to differentiate the DNA from dead cells and live cells and thus it can accurately reflect the number of live cells.

### Detection of viable *S. aureus* in milk

**Fig. 3:**
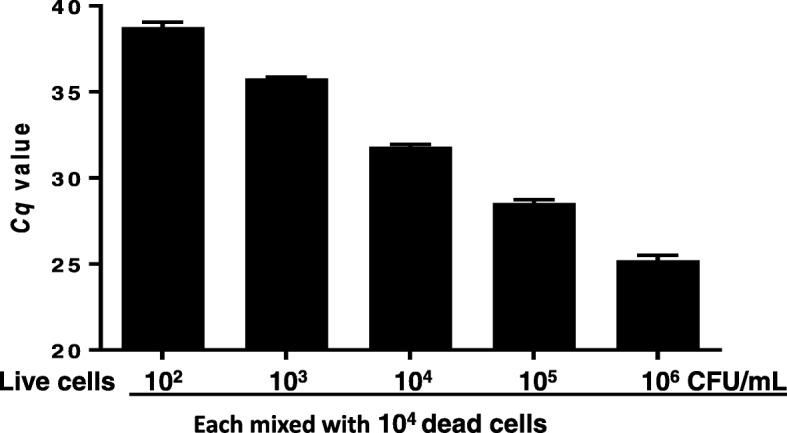
Detection of viable *S. aureus* cells in spiked milk samples by the PMA-qPCR assay. All milk samples (1 ml) were mixed with 10^4^ dead *S. aureus* cells, and then each sample was mixed with a different number of live *S. aureus* cells (10^2^, 10^3^, 10^4^, 10^5^, and 10^6^ CFU/ml), respectively. Each concentration of cell was sampled in triplicate

In the presence of a large number of dead cells (10^4^ CFU/ml), the inoculated live *S. aureus* cells (10^2^, 10^3^, 10^4^, 10^5^, and 10^6^ CFU/ml) were quantitively detected as shown in Fig. 3. The PMA-qPCR assay demonstrated that the DNA from dead cells was completely excluded from PCR amplification and the limit of detection (LOD) of the assay reached 10^2^ CFU/ml of *S. aureus* in spiked milk.

## Discussion

PCR is a useful tool for rapid detection of foodborne pathogens, however, its inability to differentiate DNA from dead cells and live cells in amplification severely limits its application in pathogen detection [7]. To overcome this drawback of PCR, a novel approach has been taken to prevent the amplification of DNA of dead cells. DNA intercalating dyes biological dies, EMA and PMA, were used to pretreat samples before DNA extraction to remove the DNA of dead cells [14, 15]. EMA can effectively penetrate dead cells and to some degree penetrate viable cells [16], whereas PMA does not significantly inhibit PCR amplification of DNA of viable cells [14, 17]. In this study, we evaluated the capability and suitability of two surfactants (triton x-100 and sarkosyl) in enhancing PMA’s permeability to dead cells to improve the efficiency of accurate detection of the DNA of live cells by incorporating treatments of PMA and surfactant in a qPCR assay.

Previously, sarkosyl has been used to increase PMA’s penetration to the dead cells and found that 0.3% sarkosyl significantly affected the viability of *S. aureus* [12]. In this study, it was also found that the viability of *S. aureus* was affected by 0.25% sarkosyl, and it can be affected more severely by higher concentrations. Comparatively, *S. aureus* cells demonstrated tolerance to a broader range of concentrations of triton x-100, suggesting triton x-100 is milder surfactant to live *S. aureus* cells and in the men time it can facilitate PMA’s penetration to the cell membranes of dead cells.

The analysis on the toxic effect of the two surfactants showed that 0.5% triton x-100 or 0.025% sarkosyl virtually did not affect the viability of the live cells. The use of 0.5% triton x-100 or 0.025% sarkosyl in the dead cells prior to PMA treatment achieved much higher *Cq* values compared to the PBS control, indicating the two surfactants helped PMA penetrate the cell membranes of the dead cells, which led to a more complete removal of DNA of the dead cells. On the other hand, when the treatment of surfactants was combined with PMA, the synergy of the treatments did show some affect to the viability of the live cells compared to the cells treated with PMA and PBS; and the treatment of PMA with sarkosyl affected live cells more severely than with triton x-100, suggesting *S. aureus* is more sensitive to the treatment of PMA with sarkosyl than PMA with triton x-100, in other words, treatment of PMA and triton x-100 was gentler to *S. aureus* cells than PMA and sarkosyl. According to the result of the three groups (0.5% triton x-100, 0.025% sarkosyl and PBS), optimized PMA treatment concentration was 40 μM, which was same used by Dong et al. [18]. Taking together, sample treatment of PMA and triton x-100 is more advantageous for dead cell DNA removal in qPCR. Hence, we chose the combination of PMA (40 μM) and triton x-100 (0.5%) for sample treatment in the detection of live *S. aureus* cells in spiked milk.

The ability of the PMA-qPCR to accurately detect live cells was validated by the application this assay to spiked milk. It showed that the assay reached the LOD of 10^2^ CFU/ml, which is compatible to a previous report, in which a qPCR assay showed a LOD of 3.0 × 10^2^ CFU/g in spiked milk powder [19]. Furthermore, in the inclusivity and exclusivity testing, the *S. aureus* strains tested (*n* = 21) were positively detected by the qPCR assay and none of the non-*S. aureus* strains tested (*n* = 30) showed cross-reactivity (Table 1). Thus, our data confirmed that the PMA-qPCR assay developed in this study is sensitive, specific, and accurate for detection of *S. aureus* in food. Also, the information on selection of appropriate surfactant for improving PMA’s dead cell DNA removal efficiency is useful, and it can be used for detection of other pathogens as well, particularly pertinent to Gram-positive pathogens.

## Conclusions

In this study, we developed a qPCR assay in conjunction with treatments of PMA and surfactants. This assay offers a sensitive and accurate means for detection of viable *S. aureus* cells. Cell toxic effect testing with the two surfactants showed that the viability of *S. aureus* was virtually not affected by the treatment with 0.5% triton x-100 or 0.025% sarkosyl. The PMA-qPCR results indicated that the assay reached high an amplification efficiency and the live *S. aureus* cells were accurately detected from the triton-treated spiked milk samples. Additionally, the information on cell sample treatment with surfactants to improve the dead cell DNA removal efficiency in qPCR by increasing PMA’s permeability to dead cells can be used for other pathogens, especially for Gram-positive bacteria.

## Abbreviations

DNA: Deoxyribonucleic acid
EMA: Ethidium monoazide
GLM: General Linear Model
LB: Luria Bertani
LOD: Limit of detection
MRSA: Methicillin-resistant *Staphylococcus aureus*
NC: Negative control
PBS: Phosphate buffered saline
PCR: Polymerase chain reaction
PMA: Propidium monoazide
WHO: World Health Organization
